# Dr. Martin E. Brackett

**Published:** 1904-03

**Authors:** 


Dr. Martin E. Brackett, of Clarendon, N. Y., died at his home in that village January 2, 1904. Dr. Brackett graduated at the University of Buffalo in 1891.

